# 

**Published:** 1860-09

**Authors:** 


**Payments.**

W. McLean, $3; R. W. Hubert, $3; H. L. Currier, $3; G. L. Hunter, $3; W. J. Swanson $3; L. Morgan, $3; W. C. Patterson, $6; L. H. Freeman, $2; H. R. Casey, $3; E. C. Hawes, $3; B. Brown, $3; W. A. Culbertson, $12; P. R. Shi, $2; Eli Frost, $5; N. W. Gable, $2; H. J. Smith, $12; W. C. Moore, $2; W. H. Monteith, $3; W. Dobbs, $3; J. J. A. Smith, $3; J. I. Cowan, $2; Daniel McBean, $10; W. Burch, $3; J. Sawyer, $3; W. A. Sparkman, $3; A. D. Rogers, $3; Jas. W. Taylor, $3; J. W. Heard, $5; Pinkston & Waldrop, $3; Henry Gaither, $5; Willis Willingham $3; C. C. Loyd, $4; Greene & Bloodworth, $3.10; Reuben Searcy, $2; Thos. Darnall, $6; W. A. Herndon, $4.50; A. M. Wilkens, $6; A. G. Cheatham, $3; R. S. Payne, $3.50; Jno. Rhea, 2, $5; J. A. Hayes, $1.50; R. C. Mayson, $2; B. A. Vaughn, $15.

### TO CORRESPONDENTS.

By a recent change in the management of the Journal, its whole business affairs will hereafter come under the supervision of the Editor, and it is therefore requested that ALL Communications, whether relating to the Editorial, Subscription, Advertising or Financial Departments, should be addressed to J. G. WESTMORELAND, or Editor of Medical and Surgical Journal, Atlanta, Ga.

☞ The Advertisement of the Winter Course in Atlanta Medical College, commencing on the First Monday in November next, is unavoidably crowded out of this number.

